# Comparative Efficacy and Safety of Immunotherapy on Non–Small Cell Lung Cancer Patients With Brain Metastases: A Systematic Review and Network Meta‐Analysis

**DOI:** 10.1111/crj.13823

**Published:** 2024-08-20

**Authors:** Tianyi Lyu, Bo Sun, Daowen Yang, Xirui Zhao, Ruoshui Wang, Xinyang Shu, Demin Li, Hong Chen

**Affiliations:** ^1^ Acupuncture Department Obstetrics and Gynecology Hospital, Capital Medical University, Beijing Maternal and Child Health Care Hospital Beijing China; ^2^ Asset Management Division Beijing University of Chinese Medicine Third Affiliated Hospital Beijing China; ^3^ National Center for Respiratory Medicine, National Clinical Research Center for Respiratory Diseases, Institute of Respiratory Medicine, Chinese Academy of Medical Sciences, Department of Traditional Chinese Medicine for Pulmonary Diseases, Center of Respiratory Medicine, China‐Japan Friendship Hospital Beijing China; ^4^ School of Acupuncture‐Moxibustion and Tuina Beijing University of Chinese Medicine Beijing China; ^5^ Surgical Department Dongfang Hospital Beijing University of Chinese Medicine Beijing China

**Keywords:** brain neoplasms, carcinoma, immunotherapy, network meta‐analysis, non–small cell lung

## Abstract

**Background:**

Growing evidence suggests that immunotherapy has a positive effect on non–small cell lung cancer (NSCLC) patients with brain metastases (BMs). However, it remains unclear which type of immunotherapy is more efficient. The aim of this network meta‐analysis (NMA) was to compare the efficacy and safety of different immunotherapy types and determine the optimal option.

**Method:**

Four databases (PubMed, Cochrane Library databases, Embase, and Web of Science) and ClinicalTrial.gov were searched from inception until January 26, 2023. Randomized controlled trials (RCTs), prospective nonrandomized trials, or observational studies investigating NSCLC patients with BMs treated by immunotherapy were included. The quality of the included studies was evaluated using the Cochrane risk of bias (ROB) tool and the Newcastle‐Ottawa Scale (NOS). The efficacy of immunotherapy on NSCLC patients with BMs was evaluated using frequentist random‐effects NMA.

**Result:**

Eleven studies from 1560 citations, encompassing 1437 participants, were included in this NMA. Statistical analysis showed that pembrolizumab (SMD = 4.35, 95% CI [2.21, 6.60]) and nivolumab+ipilimumab (SMD = 3.81, 95% CI [1.21, 6.40]) could improve overall survival (OS). Pembrolizumab (SMD = 3.32, 95% CI [2.75, 3.90]) demonstrated better effects in improving the overall response rate (ORR). No significant difference in adverse event (AE) was observed between immunotherapy and chemotherapy.

**Conclusion:**

Our findings indicated that pembrolizumab was the most promising immunotherapy for NSCLC patients with BMs. Nivolumab+ipilimumab might be an alternative choice to improve OS.

**Limitation:**

Inconsistency tests were not performed because of the scarcity of direct comparison. Besides, high heterogeneity was observed in our NMA.

## Graphical abstract

NMA was conducted to compare the efficacy and safety of different immunotherapy types on NSCLC patients with BMs. Our findings indicated that pembrolizumab was the most promising immunotherapy.

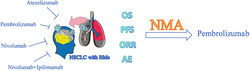

## 1 Introduction

Brain metastases (BMs) are frequent cancer complications, with an incidence rate of 10%–26% among cancer patients who die [1]. NSCLC is one of the most common tumors associated with BMs [2]. BMs were present in approximately 20% of NSCLC patients at the time of diagnosis, and another 25%–50% will develop BMs over the course of the cancer [3]. BMs are serious concerns for NSCLC patients, as they can significantly impact life expectancy [4].

BMs are therapeutic problems that need a multidisciplinary strategy to achieve timely local control [5]. Unfortunately, traditional treatments (such as surgery, radiation, and chemotherapy) only provide a marginal survival benefit, accompanied by a high incidence of neurotoxicity and high fatality rates [6, 7, 8].

Over the last several decades, the development of immunotherapy has revolutionized cancer treatment and is considered a new standard of care across many cancer indications [9]. According to the latest guidelines from ESMO and ASCO, immunotherapy is superior to chemotherapy in the treatment of lung cancer. Meanwhile, the overall incidence of adverse events (AEs) was similar between immunotherapy and chemotherapy. Growing evidence suggests that immunotherapy might have a beneficial effect on NSCLC patients with BMs [10, 11, 12]. A meta‐analysis showed that both BM and non‐BM lung cancer patients could obtain comparable benefits from immunotherapy [13]. Another previous study revealed that immunotherapy combined with chemotherapy was the best option for NSCLC patients with BM [14]. However, none of the previous studies compared the differences in efficacy and safety among different types of immunotherapy in the treatment of NSCLC with BM [15, 16].

Although many types of immunotherapy (e.g., pembrolizumab, nivolumab, and atezolizumab) have been proven to be effective and safe, physicians are still facing the problem of making clinical decisions on which type of immunotherapy to choose when treating NSCLC patients with BM [17, 18, 19]. Therefore, we designed and conducted this NMA to provide an up‐to‐date analysis to compare efficacy and safety among different immunotherapy types and provide insights into the optimal immunotherapy to inform clinical decision‐making.

## 2 Methods

### 2.1 Search Strategy and Study Selection

The NMA was reported in accordance with the PRISMA statement [20], and the study protocol was registered on PROSPERO (CRD42023403657). Four databases (PubMed, Embase, Cochrane Library, and Web of Science) and ClinicalTrial.gov were searched from inception until January 26, 2023, and the search strategy is described in Table S1.

The PICOS (participants, interventions, comparators, outcomes, and study design) approach was applied to determine inclusion criteria [21]. Studies had to fulfill the following criteria to be considered for inclusion: (1) population: NSCLC patients with BMs; (2) intervention: immunotherapy combined with chemotherapy; (3) comparison: chemotherapy; (4) outcome: overall survival (OS), progression‐free survival (PFS), overall response rate (ORR), and AE; (5) study design: randomized controlled trials, prospective nonrandomized trials, or observational studies (prospective or retrospective).

The exclusion criteria were as follows: (1) not providing adequate information to assess the effect size; (2) reviews, editorials, comments, case reports, animal trials, letters, and study protocol; (3) the type of intervention being unclear; (4) articles without a control group; (5) articles without immunotherapy; (6) articles without outcomes on BMs.

### 2.2 Data Extraction and Processing

Two researchers (RS‐W and XR‐Z) independently and blindly searched databases for relevant references and deleted duplicate entries, with disagreements judged by a third researcher (TY‐L). After deleting duplicate entries, two researchers (RS‐W and XR‐Z) independently and blindly screened articles and extracted data from these articles, with disagreements judged by a third researcher (TY‐L). In addition, we also extracted baseline characteristics (name of the first author, year of publication, type of study, country of origin, gender trends, mean age, status of CNS symptom, use of steroid, intervention type, and outcomes).

### 2.3 Outcomes

The main outcome data included PFS (the time from randomization to objective tumor progression), OS (the time from randomization to all‐cause death), ORR (the sum of the proportion of patients getting a complete intracranial response), and AEs. The mean and standard deviation were applied as the units of analysis for continuous outcomes. The number of participants with events and the total number of participants were applied as the units of analysis for dichotomous outcomes.

### 2.4 Assessment of Heterogeneity and Inconsistency

The *I* ^2^ statistic, as mentioned in Section 9.5.2 of the Cochrane Handbook for Systematic Reviews of Interventions, was applied to assess heterogeneity. Based on this section, we assessed the *I* ^2^ statistic using the following criteria: 0%–40%: might not be important; 30%–60%: may represent moderate heterogeneity; 50%–90%: may represent substantial heterogeneity; 75%–100%: considerable heterogeneity [22]. We failed to perform inconsistency tests because the closed loops were not formed. All heterogeneity analyses were performed with the statistical software R Version 3.2.2.

### 2.5 Metaregression

We hypothesized that study type (RCT or retrospective study) and treatment line (no previous systemic therapies or undergoing at least first‐line treatment) were potential covariates contributing to heterogeneity [14, 23]. Therefore, we performed metaregression to assess the association between these covariates and the treatment effects if the heterogeneity was substantial or considerable. Given that the range between 2.5%β and 97.5%β covered 0, we concluded that the covariate significantly contributed to the heterogeneity. All metaregressions were performed with the statistical software R Version 3.2.2.

### 2.6 Quality Assessment

Two researchers (RS‐W and XR‐Z) independently and blindly evaluated the risk of bias of the included RCTs using the Cochrane risk of bias tool for randomized trials and the quality of the included observational studies using the Newcastle‐Ottawa Scale (NOS) [24, 25]. The results were submitted to the Confidence in Network Meta‐Analysis (CINeMA) tool, which was used to assess the credibility of each NMA's findings. CINeMA grade confidence levels were classified as high, moderate, low, or very low [26].

### 2.7 Sensitivity Analysis

We conducted sensitivity analysis by repeating each NMA after removing studies with a high overall risk of bias or a small sample size (< 30) [27].

### 2.8 Statistical Analysis

First, we integrated each type of outcome by network graph. Second, all network comparisons were assessed by random‐effects frequentist NMA. Standard mean differences (SMDs) with 95% confidence intervals (CIs) were utilized to assess outcomes for each parameter and treatment comparison. In all forest plots, chemotherapy was utilized as the reference group. Third, the league tables were conducted to show the relative degree of outcomes for all comparisons among immunotherapies. *p*‐scores were utilized to rank immunotherapy based on outcomes. *p*‐scores were given a value between zero and one, with a higher *p*‐score indicating a larger effect. Fourth, a comparison‐adjusted funnel plot and Egger's test were performed to evaluate the publication bias, with Egger's test indicating publication bias when *p* < 0.05. Fifth, the status of CNS symptoms and the use of steroid treatment were stratified to determine their impact on immunotherapy efficacy. After summarizing all 11 included articles, we found that (1) in terms of the status of CNS symptoms, nine studies were conducted in asymptomatic BMs and two did not report it. Based on these findings, we conducted each NMA on studies with asymptomatic BMs. (2) In terms of steroid treatment, six studies included patients who did not receive steroids, one included patients who received steroids, and four did not report it. Accordingly, we conducted each NMA on studies with patients who did not receive steroids. Finally, StataSE (Version 16) was used to conduct network plots, comparison‐adjusted funnel plots, and Egger's test. Statistical software R (Version 3.2.2) was used to conduct other NMAs.

## 3 Results

### 3.1 Literature Search and Characteristics of the Included Studies

**FIGURE 1:**
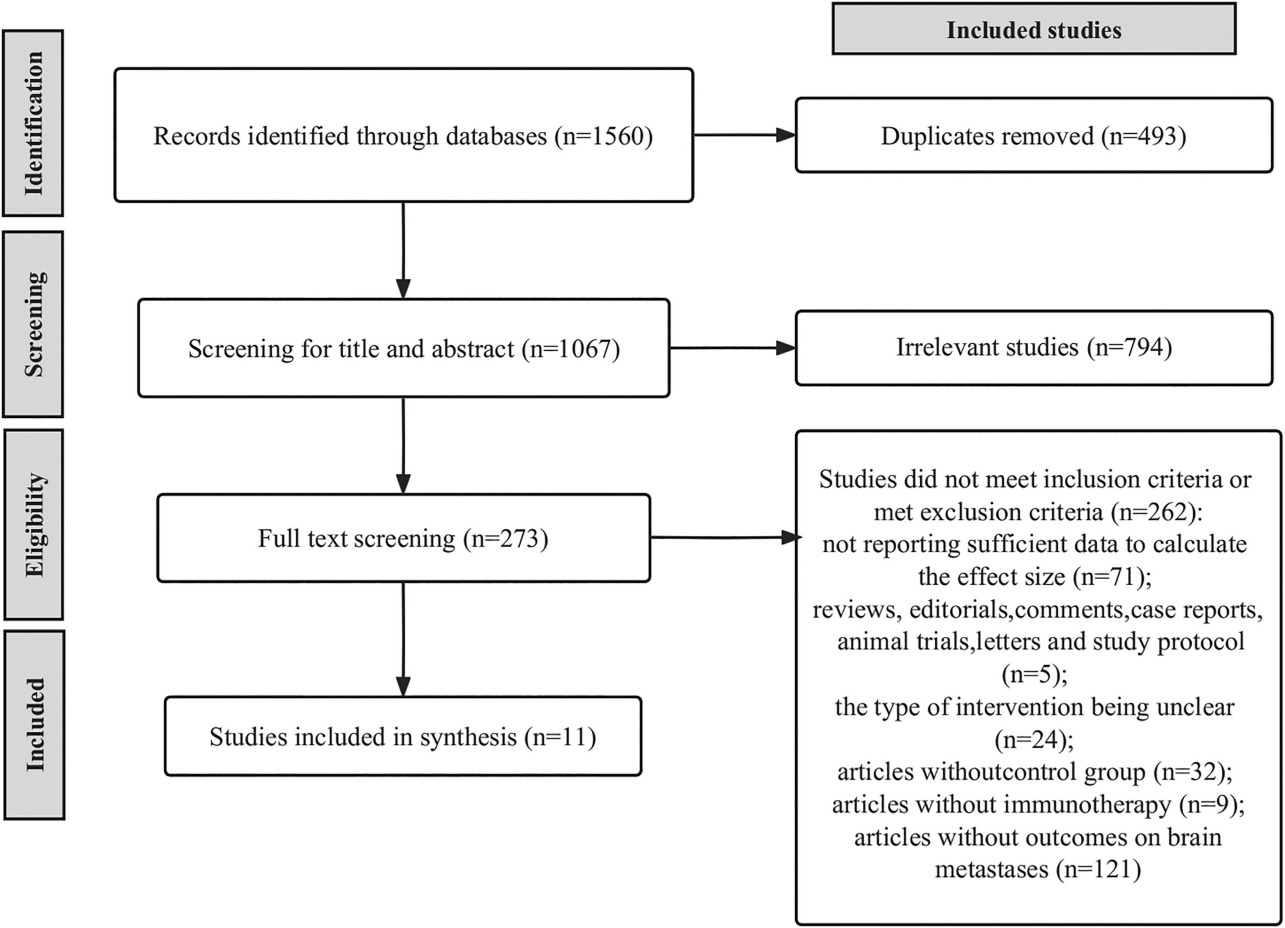
PRISMA flowchart illustrating the selection of studies included in our analysis.

After removing duplicated articles, 1560 relevant studies were identified through the literature search. Two hundred seventy‐three articles were identified after scanning titles and abstracts. Two hundred sixty‐two articles were removed after full‐text reading for the following reasons: not providing adequate information to assess the effect size (*n* = 71); reviews, editorials, comments, case reports, animal trials, letters, and study protocol (*n* = 5); the type of intervention being unclear (*n* = 24); articles without a control group (*n* = 32); articles without immunotherapy (*n* = 9); and articles without outcomes on BMs (*n* = 121). Finally, we identified 11 eligible RCTs (Figure 1) [28, 29, 30, 31, 32, 33, 34, 35, 36, 37, 38], containing 1437 participants, 860 (59.8%) of whom received immunotherapy and 577 (40.2%) received control intervention. The baseline characteristics of the included studies are summarized in Table S2.

### 3.2 Risk of Bias

To assess the risk of bias in the six included RCTs (Table S3), the Cochrane Risk of Bias tool was applied. To assess the quality of five retrospective studies (Table S4), NOS was applied.

**FIGURE 2:**
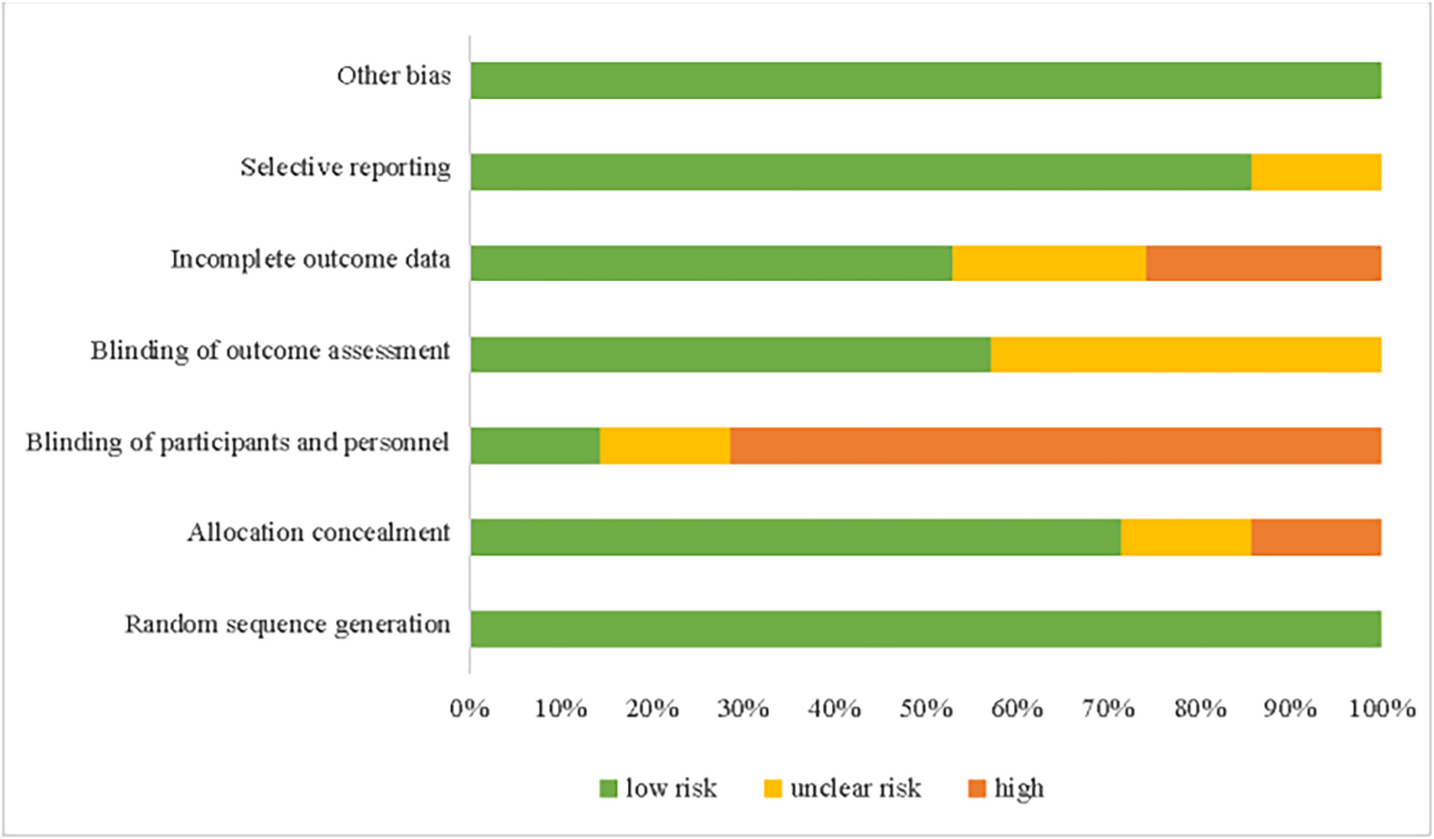
Risk of bias of RCTs.

The Cochrane risk of bias assessment showed that one (16.7%) study had a low risk of bias, four (66.6%) had a moderate risk of bias, and one (16.7%) had a high risk of bias (Figure 2 and Table S3). For random sequence generation, six (100%) studies had low risk of bias; for allocation concealment, one (16.7%) study was unclear and one (16.7%) at high risk of bias; for blinding of participants and personnel, one (16.7%) study was unclear and four (66.7%) at high risk of bias; for blinding of outcome assessment, three (50%) studies were unclear and three (50%) at low risk of bias; for incomplete outcome data, two (33.3%) studies were unclear and four (66%) at low risk of bias; for selective reporting, one (16.7%) study was unclear and five (83.3%) at low risk of bias; and for other bias, six studies were at low risk of bias.

The NOS quality assessment showed that four (80%) studies had a low risk of bias and one (20%) had a moderate risk of bias (Table S4). For the selection of cases and controls, three (60%) studies scored 4 points and two (40%) scored 3 points. For comparability of cases and controls, four (80%) studies scored 1 point and one (20%) scored 0 points. For exposure, five (100%) studies scored 3 points.

### 3.3 OS

**FIGURE 3:**
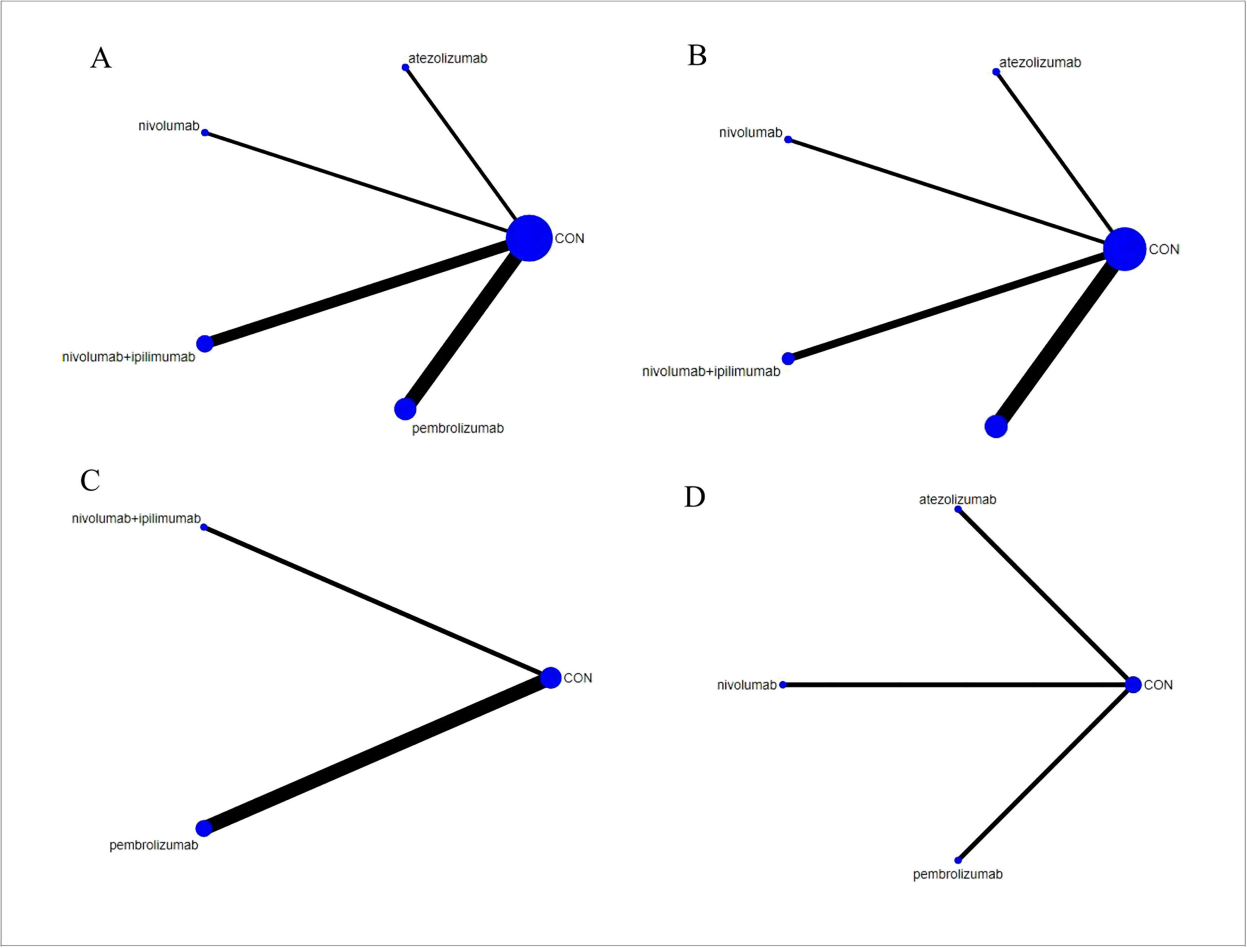
Network plots of the outcomes of the comparisons between immunotherapy and controls in the NMA. (A) OR; (B) PFS; (C) ORR; (D) AE. Treatments with direct comparisons are linked with a line; the thickness of connecting lines corresponds to the number of trials evaluating the comparison; the size of the circle is proportional to the sample size. CON, chemotherapy.

**FIGURE 4:**
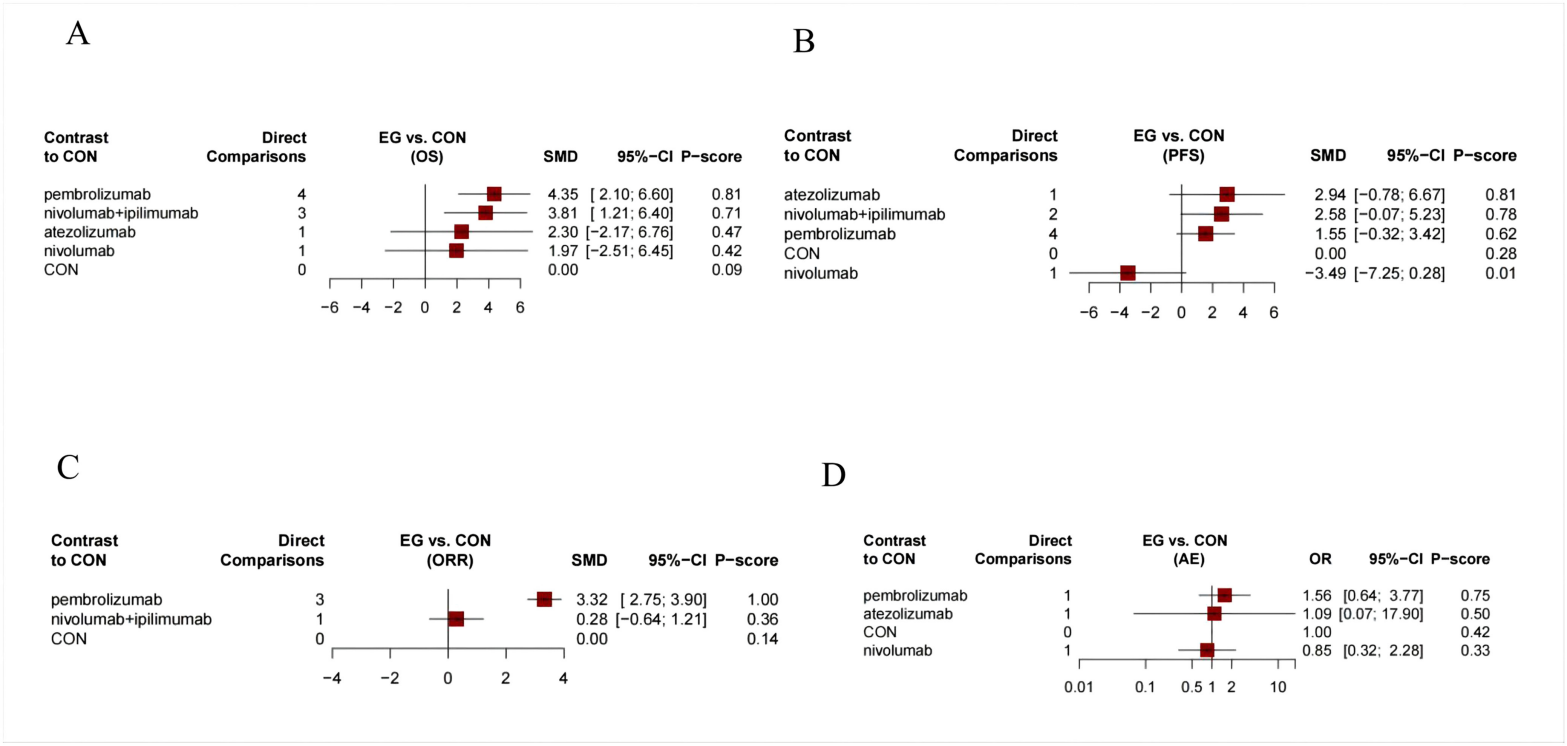
Forest plots of the efficiency of comparisons between immunotherapy and control. (A) OS; (B) PFS; (C) ORR; (D) AE. 95% CI > 0 means the intervention group is superior to the control group significantly. *p*‐scores were applied to rank gait training on the basis of balance outcome. *p*‐scores ranged from 0 to 1, and a higher *p*‐score indicates a greater effect. CON, chemotherapy.

As shown in Figure 3A, the OS outcome was reported in nine studies, comparing four types of immunotherapy (661 patients) with chemotherapy (483 patients). Our results demonstrated that pembrolizumab and nivolumab+ipilimumab were significantly more effective than chemotherapy (Figure 4A). Based on the *p*‐score, pembrolizumab was considered the most effective, while nivolumab was the least effective (Figure 4A). We did not find any significant difference among immunotherapy types (Table 1). The *I* ^2^ was 97.8%. Due to the lack of direct comparisons, we could not observe inconsistencies between direct and indirect comparisons. In all comparisons, CINeMA grade confidence was low or very low (Table S5A). In addition, no publication bias was observed in OS NMA (Egger's test: *p* = 0.135, Figure S1A). In patients with asymptomatic BMs, the overall findings remained the same (Figure S5A). In patients who did not receive the steroid treatment, only pembrolizumab appeared significantly superior to the control intervention (Figure S6A).

**TABLE 1:** The league table for OS estimate intervention according to their relative effects and 95% credibility intervals (95% CI).

Atezolizumab	2.30 (−2.17; 6.76)	NA	NA	NA
2.30 (−2.17; 6.76)	CON	−1.97 (−6.45; 2.51)	−3.81 (−6.40; −1.21)	−4.35 (−6.60; −2.10)
0.33 (−6.00; 6.65)	−1.97 (−6.45; 2.51)	Nivolumab	NA	NA
−1.51 (−6.68; 3.65)	−3.81 (−6.40; −1.21)	−1.84 (−7.02; 3.34)	Nivolumab+ipilimumab	NA
−2.05 (−7.05; 2.95)	−4.35 (−6.60; −2.10)	−2.38 (−7.39; 2.64)	−0.54 (−3.98; 2.89)	Pembrolizumab

Abbreviation: CON, chemotherapy.

### 3.4 PFS

As shown in Figure 3B, the PFS outcome was reported in eight studies, comparing four types of immunotherapy (410 patients) with chemotherapy (336 patients). None of the included immunotherapies demonstrated any significant effect when compared to chemotherapy (Figure 4B). Based on the *p*‐score, atezolizumab was considered the most effective, while nivolumab was the least effective (Figure 4B). We did not find any significant difference among immunotherapy types (Table 2). The *I* ^2^ was 97.7%. Due to the lack of direct comparisons, we could not observe inconsistencies between direct and indirect comparisons. In all comparisons, CINeMA grade confidence was low or very low (Table S5B). In addition, no publication bias was observed in PFS NMA (Egger's test: *p* = 0.534, Figure S1B). In patients with asymptomatic BMs, the overall findings remained the same (Figure S5B). In patients who did not receive the steroid treatment, both atezolizumab and pembrolizumab appeared significantly superior to the control intervention (Figure S6B).

**TABLE 2:** The league table for PFS estimate intervention according to their relative effects and 95% credibility intervals (95% CI).

Atezolizumab	2.94 (−0.78; 6.67)	NA	NA	NA
2.94 (−0.78; 6.67)	CON	3.49 (−0.28; 7.25)	−2.58 (−5.23; 0.07)	−1.55 (−3.42; 0.32)
6.43 (1.13; 11.73)	3.49 (−0.28; 7.25)	Nivolumab	NA	NA
0.36 (−4.21; 4.93)	−2.58 (−5.23; 0.07)	−6.07 (−10.67; −1.46)	Nivolumab+ipilimumab	NA
1.39 (−2.78; 5.56)	−1.55 (−3.42; 0.32)	−5.04 (−9.25; −0.83)	1.03 (−2.22; 4.27)	Pembrolizumab

Abbreviation: CON, chemotherapy.

### 3.5 ORR

As shown in Figure 3C, the ORR outcome was reported in four studies, comparing two types of immunotherapy (382 patients) with chemotherapy (216 patients). Our results demonstrated that only pembrolizumab was significantly more effective than chemotherapy (Figure 4C). Based on the *p*‐score, pembrolizumab was considered the most effective, while nivolumab+ipilimumab was the least effective (Figure 4C). We did not find any significant differences among immunotherapy types (Table 3). The *I* ^2^ was 72.1%. Due to the lack of direct comparisons, we could not observe inconsistencies between direct and indirect comparisons. In all comparisons, CINeMA grade confidence was low or very low (Table S5C). In addition, no publication bias was observed in ORR NMA (Egger's test: *p* = 0.651, Figure S1C). In patients with asymptomatic BMs or patients who did not receive the steroid treatment, the overall findings remained the same (Figures S5C and S6C).

**TABLE 3:** The league table for ORR estimate intervention according to their relative effects and 95% credibility intervals (95% CI).

CON	−0.28 (−1.21; 0.64)	−3.32 (−3.90; −2.75)
−0.28 (−1.21; 0.64)	Nivolumab+ipilimumab	NA
−3.32 (−3.90; −2.75)	−3.04 (−4.13; −1.94)	Pembrolizumab

Abbreviation: CON, chemotherapy.

### 3.6 AE

As shown in Figure 3D, the AE outcome was reported in three studies, comparing three types of immunotherapy (191 patients) with chemotherapy (160 patients). None of the included immunotherapies demonstrated any significant adverse effects when compared to chemotherapy (Figure 4D). We did not find any significant differences among immunotherapy types (Table 4). We failed to conduct heterogeneity and inconsistency tests due to the scarcity of comparisons. In all comparisons, CINeMA grade confidence was low or very low. In addition, no publication bias was observed in AE NMA (Egger's test: *p* = 0.883, Figure S1D). In patients with asymptomatic BMs or patients who did not receive the steroid treatment, the overall findings remained the same (Figures S5D and S6D).

**TABLE 4:** The league table for AE estimate intervention according to their relative effects and 95% credibility intervals (95% CI).

Atezolizumab	1.09 (0.07; 17.90)	NA	NA
1.09 (0.07; 17.90)	CON	1.18 (0.44; 3.17)	0.64 (0.26; 1.56)
1.29 (0.07; 25.01)	1.18 (0.44; 3.17)	Nivolumab	NA
0.70 (0.04; 13.19)	0.64 (0.26; 1.56)	0.55 (0.14; 2.06)	Pembrolizumab

Abbreviation: CON, chemotherapy.

### 3.7 Sensitivity Analysis

Sensitivity analysis was conducted for all four outcomes by repeating each NMA after removing one study with a small sample size and one study with low quality. The findings were essentially the same in all sensitivity analyses (Figure S2), suggesting that the removal of these studies had no significant effect on the results.

### 3.8 Metaregression

We performed metaregression for OS and PFS but not for ORR and AE due to a scarcity of studies. Our results demonstrated that study type (OS: 2.5%β = −8.409, 97.5%β = 2.046; PFS: 2.5%β = −6.472, 97.5%β = 3.498) and treatment line (OS: 2.5%β = −11.2049, 97.5%β = 37.663; PFS: 2.5%β = −19.486, 97.5%β = 11.845) did not significantly contribute to heterogeneity (Tables S6 and S7) NMA. Moreover, findings were approximately the same after repeating NMA with adjusted covariate value (Figures S3 and S4).

## 4 Discussion

Previous studies suggested that immunotherapy had a beneficial effect on NSCLC patients with BMs [10, 11, 12]. However, it remains unclear which type of immunotherapy is more effective. Therefore, the purpose of this study was to summarize the relevant literature to compare the efficacy and safety of different immunotherapy types in NSCLC patients with BMs. To evaluate the most promising immunotherapy, we conducted a NMA to combine data from 11 studies, including four different immunotherapy types, with 1437 participants. Our main findings indicated that pembrolizumab was the optimal immunotherapy to improve OS and ORR. Besides, no significant difference in AE was observed among different types of immunotherapy.

First, pembrolizumab may be the optimal option for NSCLC patients with BMs, with a longer OS and a higher ORR. Pembrolizumab is a common type of immune checkpoint inhibitor, whose core mechanism is to eliminate tumor cell inhibition of T cells [39]. Hence, whether or not pembrolizumab has an effect on BMs depends on whether or not T cells can infiltrate the malignant cells of the brain tissue. Previous studies have shown that increased T cell concentrations improve OS consistency in BMs, supporting the implementation of pembrolizumab for treating cerebral metastases [40, 41]. Our findings correspond with prior studies investigating the efficacy of pembrolizumab in NSCLC patients with BMs [42]. In terms of improving OS, we observed that nivolumab+ipilimumab was the second‐best immunotherapy. Therefore, nivolumab+ipilimumab might be an alternative choice for NSCLC patients with BMs when pembrolizumab is not suitable.

Second, there was no significant difference in PFS between immunotherapy and chemotherapy, as reported in some but not all previous studies [43, 44, 45, 46]. The inconsistencies between our findings and those previously reported might be explained by the fact that immunotherapy might only show a positive effect on PFS in specific populations. For example, a clinical trial demonstrated immunotherapy showed a significant effect on a subset of advanced NSCLC patients with PD‐L1 ≥ 50% [32, 45]. Another retrospective study suggested that PFS was improved only in the EGFR wild‐type subgroup [30].

Immunotherapy is a promising therapeutic option for NSCLC patients with cerebral metastases, but concerns exist about whether immunotherapy‐treated patients with BMs are particularly likely to suffer any treatment‐related AEs [47]. According to a published NMA, the overall incidence of AEs was similar in immunotherapy when compared with chemotherapy [15]. Another meta‐analysis suggested that immunotherapy had similar neurological AEs when compared with brain radiotherapy [13]. Meanwhile, our findings are consistent with these studies and show that the incidence of AEs is similar between immunotherapy and chemotherapy.

After stratified analysis, our findings showed that the use of steroids might be an important factor that affected immunotherapy efficacy, especially in prolonging PFS. Both atezolizumab and pembrolizumab significantly improved PFS in patients who did not receive the steroid treatment, while no significant differences were found in the overall PFS analysis. One possible explanation was that steroids could alleviate cerebral edema and decrease intracranial pressure secondary to BMs, thus masking the efficacy of immunotherapy when both the immunotherapy group and the control group were administered steroids simultaneously. The number of BMs and tumor size are also important factors to consider when addressing BM treatment. However, we failed to conduct a stratified analysis due to a lack of data. Future studies are required to collect these data to allow a more comprehensive analysis.

This study has several strengths. First, our study was the first NMA to compare the efficacy and safety of different immunotherapy regimens for NSCLS patients with BMs. Second, no evidence of publication bias was observed in all NMA analyses. Third, sensitivity analysis revealed outcomes similar to the overall findings, supporting the robustness of these results.

Our study has several limitations. First, some of the included studies were retrospective studies. Second, we failed to perform inconsistency tests because of the scarcity of direct comparisons among different immunotherapy types. Third, high heterogeneity was observed in our NMA, and our metaregression revealed that study type and treatment line did not significantly contribute to this heterogeneity. However, the results were similar to the overall findings after repeating NMA with an adjusted covariate value, suggesting the robustness of these findings. Fourth, since only one of the included studies reported intracranial outcomes, there was insufficient data to analyze the intracranial efficacy; hence, the consistency between intracranial and extracranial indicators cannot be confirmed. Fifth, the dose of steroids and the number and size of BMs are important factors that affect the therapy of BMs. After summarizing all 11 included articles, we found that (1) only one study reported the dose of steroids and the number of BMs, and (2) none of the studies reported the size of BMs. Therefore, we failed to conduct stratified analyses according to these important factors. Further research should focus on these factors and provide more comprehensive evidence for clinical practice.

## 5 Conclusion

This NMA demonstrated that pembrolizumab is the optimal option to improve OS and ORR, while nivolumab+ipilimumab was an alternative choice to improve OS. Moreover, atezolizumab and pembrolizumab had the potential to improve PFS in patients who did not receive steroid treatment. This study can help clinicians formulate better clinical decisions. However, large‐scale, high‐quality, and head‐to‐head RCTs are still needed to confirm those findings.

## Author Contributions

Conceptualization: T.Y.L., D.M.L., and H.C. Methodology: T.Y.L., B.S., and D.W.Y. Formal analysis: X.Y.S. and D.M.L. Investigation: T.Y.L. and H.C. Resources: X.R.Z. and R.S.W. Data curation: X.R.Z. and R.S.W. Writing–original draft preparation and editing: T.Y.L. and B.S. Writing–review D.M.L. and H.C. Visualization: X.R.Z. and R.S.W. Supervision: D.M.L. and H.C. All authors have read and agreed to the published version of the manuscript.

## Ethics Statement

All the included studies were approved the ethical review.

## Conflicts of Interest

The authors declare no conflicts of interest.

## Supporting information


**Figure S1** Comparison‐adjusted funnel plot.

**Figure S2** Sensitivity analysis.

**Figure S3** Forest plots of OS modified by covariate value: (A) study type; (B) treatment line. 95% CI > 0 means the intervention group is superior to the control group significantly. CON, chemotherapy.

**Figure S4** Forest plots of PFS modified by covariate value: (A) study type; (B) treatment line. 95%‐CI > 0 means the intervention group is superior to the control group significantly. CON, chemotherapy.

**Figure S5** Forest plots of the efficiency of comparisons between immunotherapy and controls in asymptomatic BMs. (A) OS; (B) PFS; (C) ORR; (D) AE. 95% CI > 0 means the intervention group is superior to the control group significantly. *p*‐scores were applied to rank gait training on the basis of balance outcome. *p*‐comes ranged from 0 to 1, a higher *p*‐score indicating a greater effect. CON, chemotherapy.

**Figure S6** Forest plots of the efficiency of comparisons between immunotherapy and controls in patients receiving steroid treatment. (A) OS; (B) PFS; (C) ORR; (D) AE. 95% CI > 0 means the intervention group is superior to the control group significantly. *p*‐scores were applied to rank gait training on the basis of balance outcome. *p*‐comes ranged from 0 to 1, a higher *p*‐score indicating a greater effect. CON, chemotherapy.

**Table S1** Search strategy for Embase.
**Table S2.** Baseline characteristics of included studies.
**Table S3.** Risk of bias graph of the included studies in this NMA.
**Table S4.** The Newcastle‐Ottawa Scale (NOS) quality assessment of the included studies in this NMA (details).
**Table S5A.** CINeMA confidence rating for OS.
**Table S5B.** CINeMA confidence rating for PFS.
**Table S5C.** CINeMA confidence rating for ORR.
**Table S5D.** CINeMA confidence rating for AE.
**Table S6A.** Metaregression of OS by study type.
**Table S6B.** Metaregression of OS by treatment line.
**Table S7A.** Metaregression of PFS by study type.
**Table S7B.** Metaregression of PFS by treatment line.

## Data Availability

The data that support the findings of this study are available from the corresponding author upon reasonable request.
