# Supplementary figures and images for: Comparative Efficacy and Safety of Immunotherapy on Non–Small Cell Lung Cancer Patients With Brain Metastases: A Systematic Review and Network Meta‐Analysis
Source: Clin Respir J. 2024 Aug 20;18(8):e13823. doi: 10.1111/crj.13823 (PMC11333852; doi:10.1111/crj.13823)

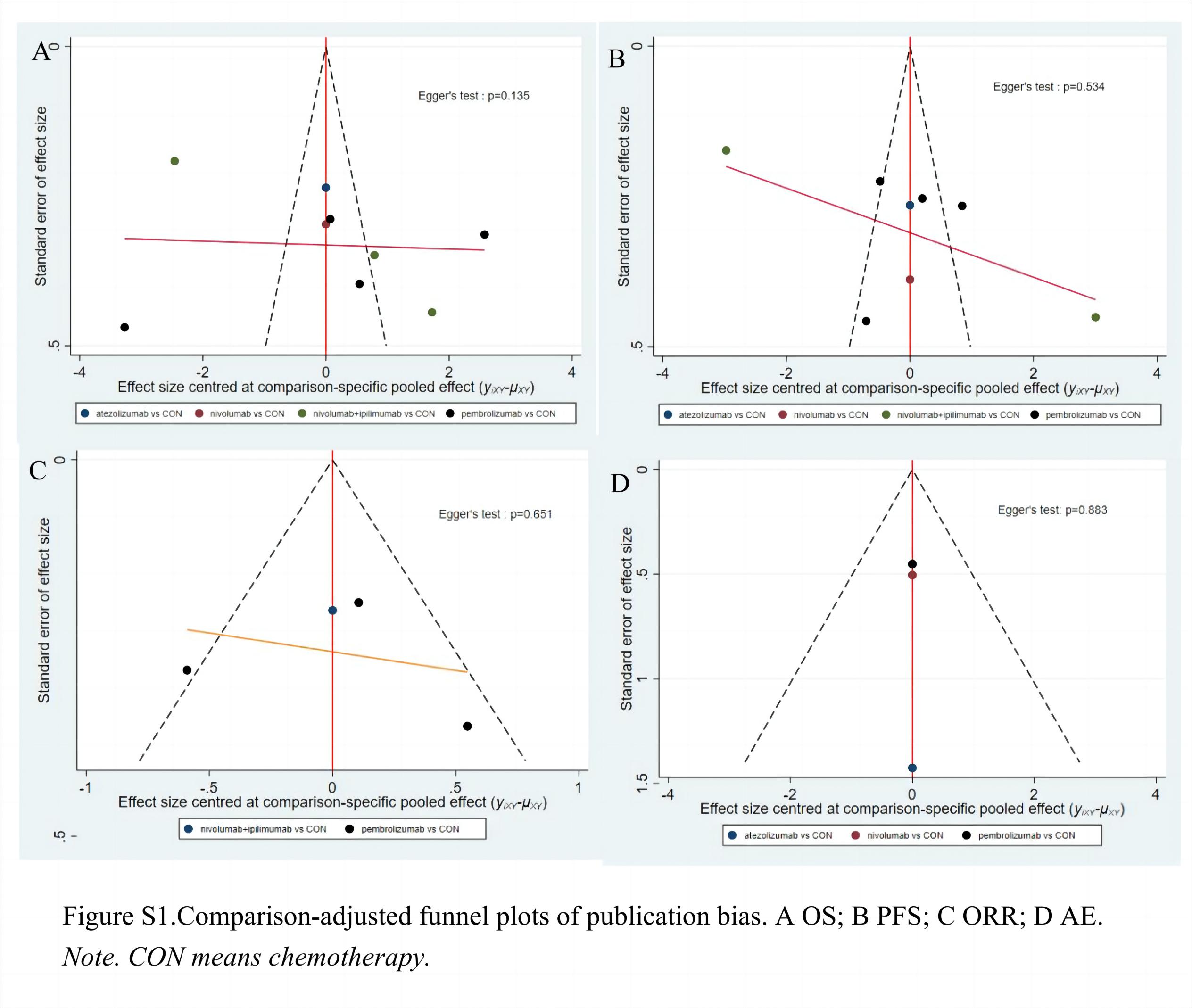

Supplement: Supplementary file 1 — Figure S1 Comparison‐adjusted funnel plot. [file CRJ-18-e13823-s005.jpg]

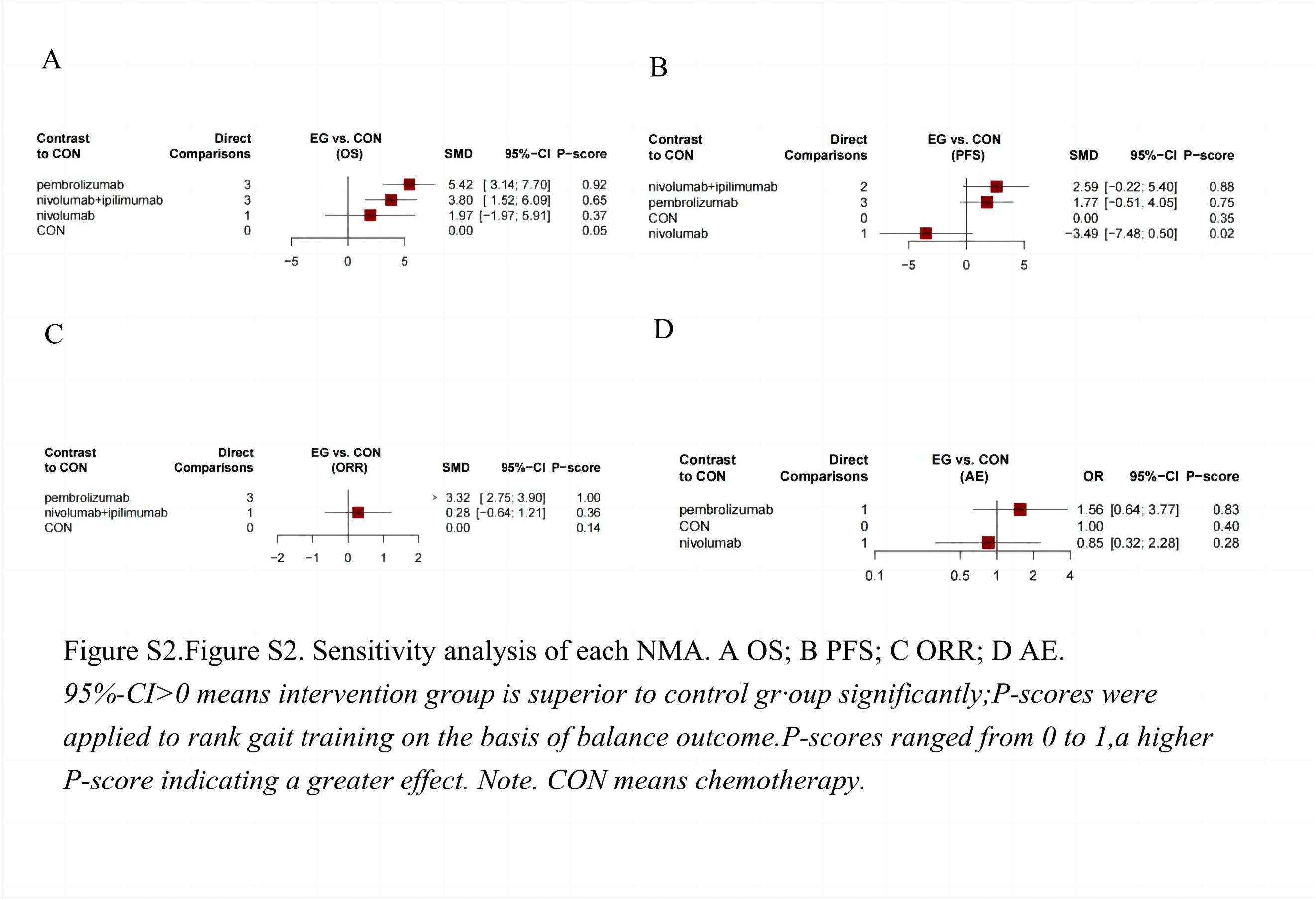

Supplement: Supplementary file 2 — Figure S2 Sensitivity analysis. [file CRJ-18-e13823-s004.jpg]

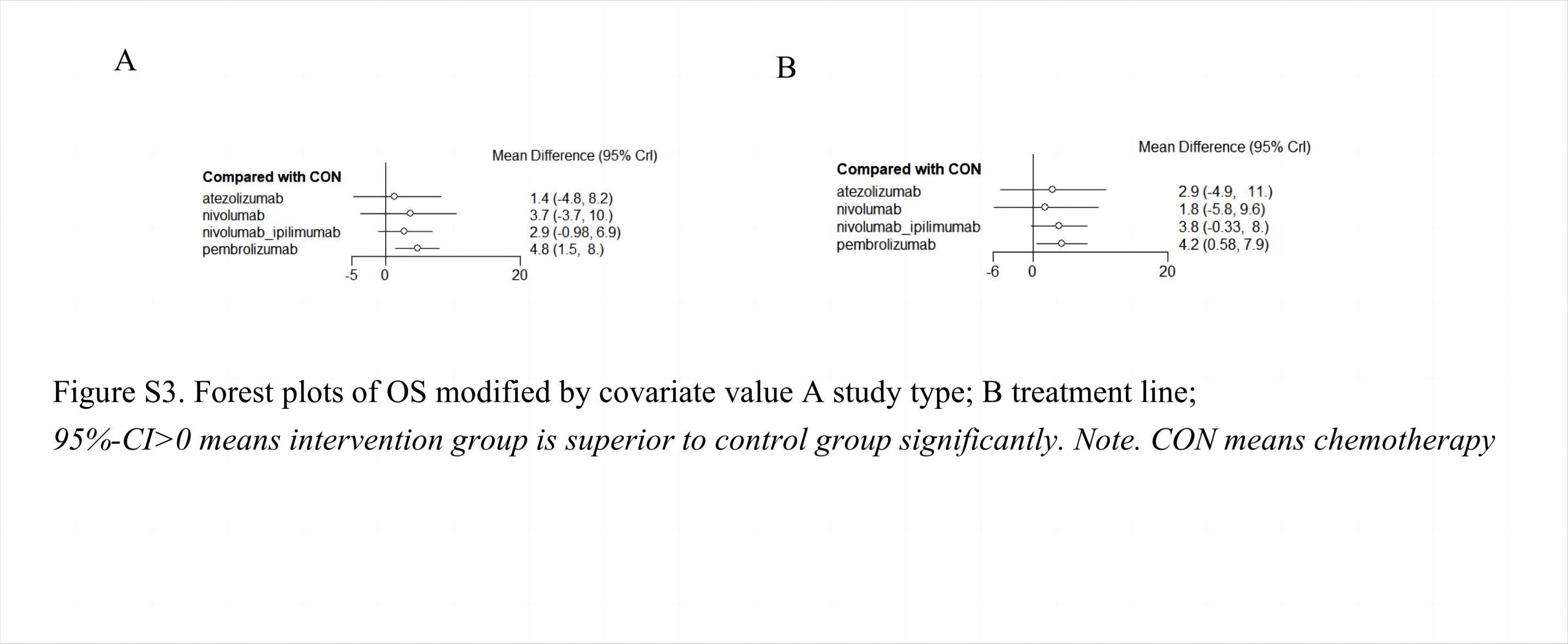

Supplement: Supplementary file 3 — Figure S3 Forest plots of OS modified by covariate value: (A) study type; (B) treatment line. 95% CI > 0 means the intervention group is superior to the control group significantly. CON, chemotherapy. [file CRJ-18-e13823-s006.jpg]

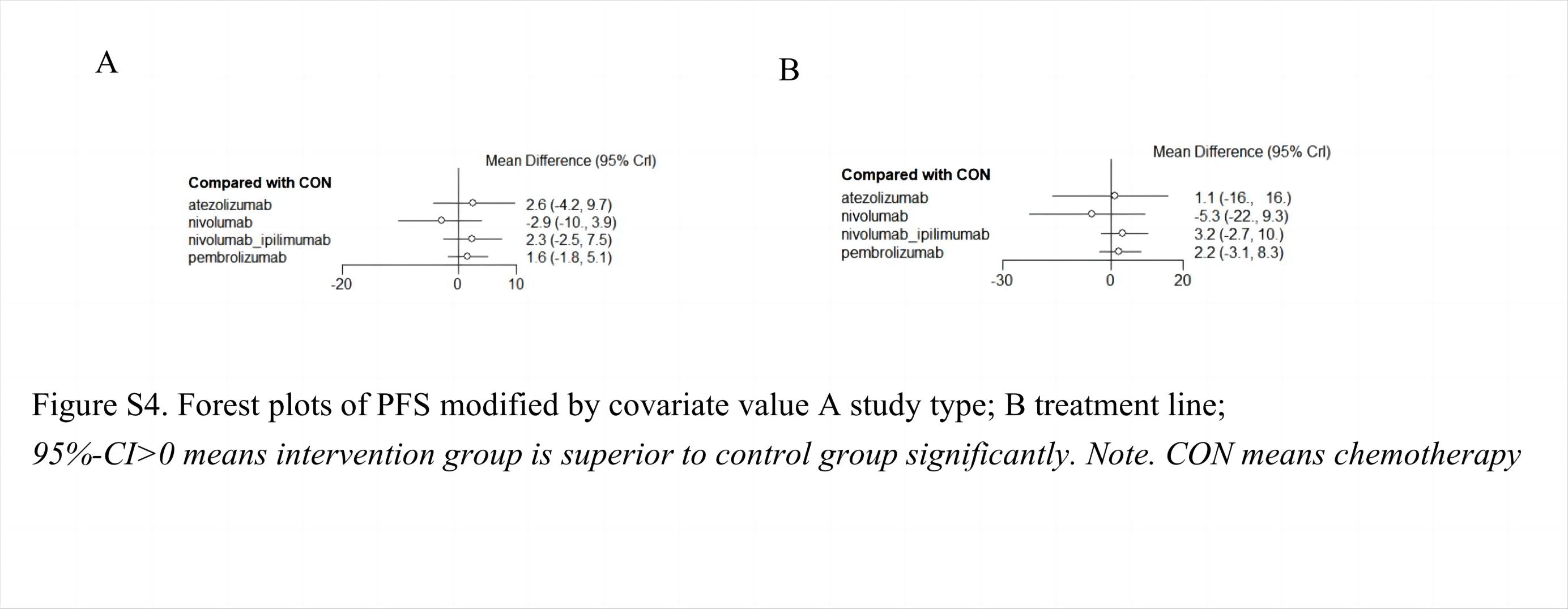

Supplement: Supplementary file 4 — Figure S4 Forest plots of PFS modified by covariate value: (A) study type; (B) treatment line. 95%‐CI > 0 means the intervention group is superior to the control group significantly. CON, chemotherapy. [file CRJ-18-e13823-s007.jpg]

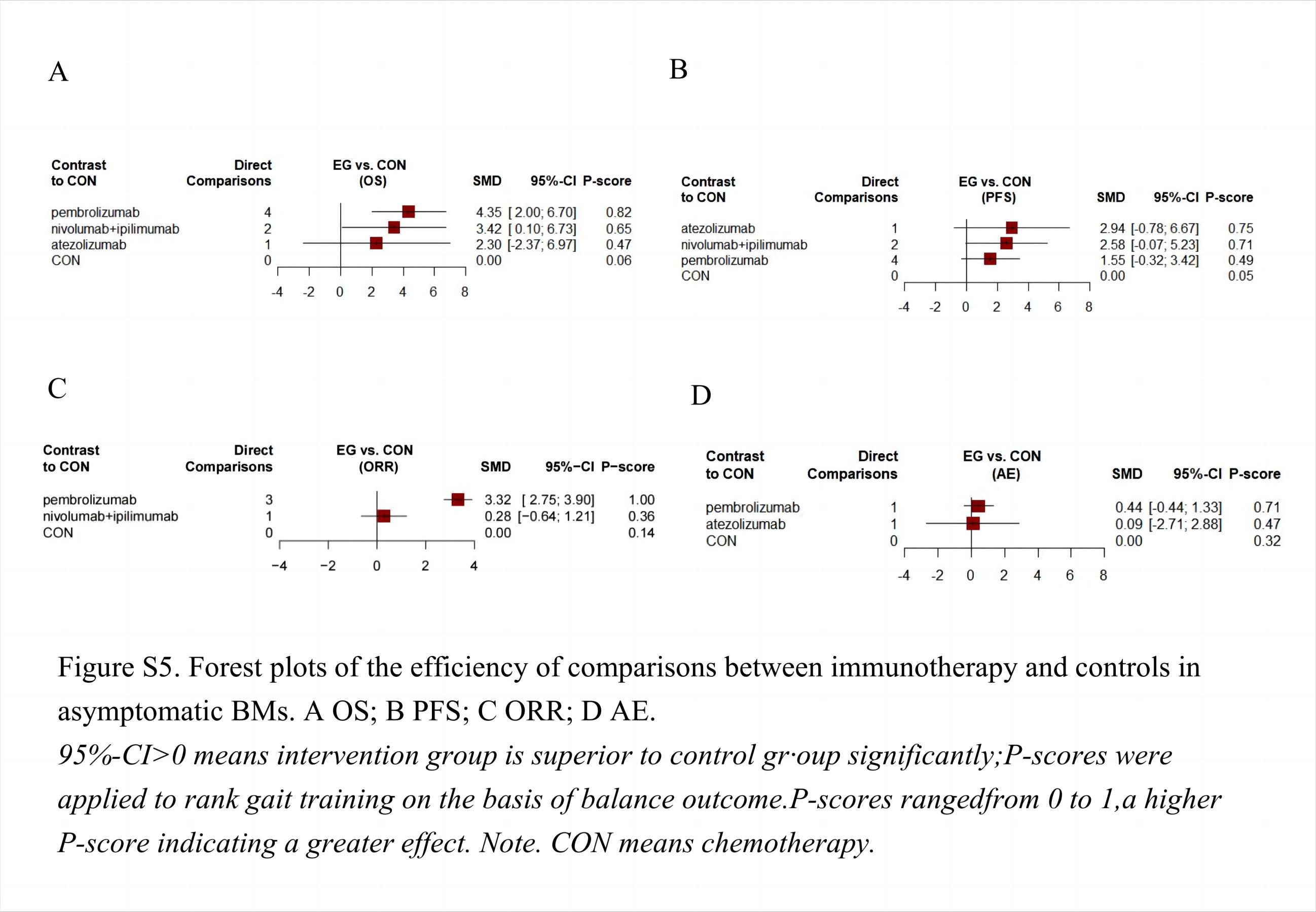

Supplement: Supplementary file 5 — Figure S5 Forest plots of the efficiency of comparisons between immunotherapy and controls in asymptomatic BMs. (A) OS; (B) PFS; (C) ORR; (D) AE. 95% CI > 0 means the intervention group is superior to the control group significantly. p‐scores were applied to rank gait training on the basis of balance outcome. p‐comes ranged from 0 to 1, a higher p‐score indicating a greater effect. CON, chemotherapy. [file CRJ-18-e13823-s003.jpg]

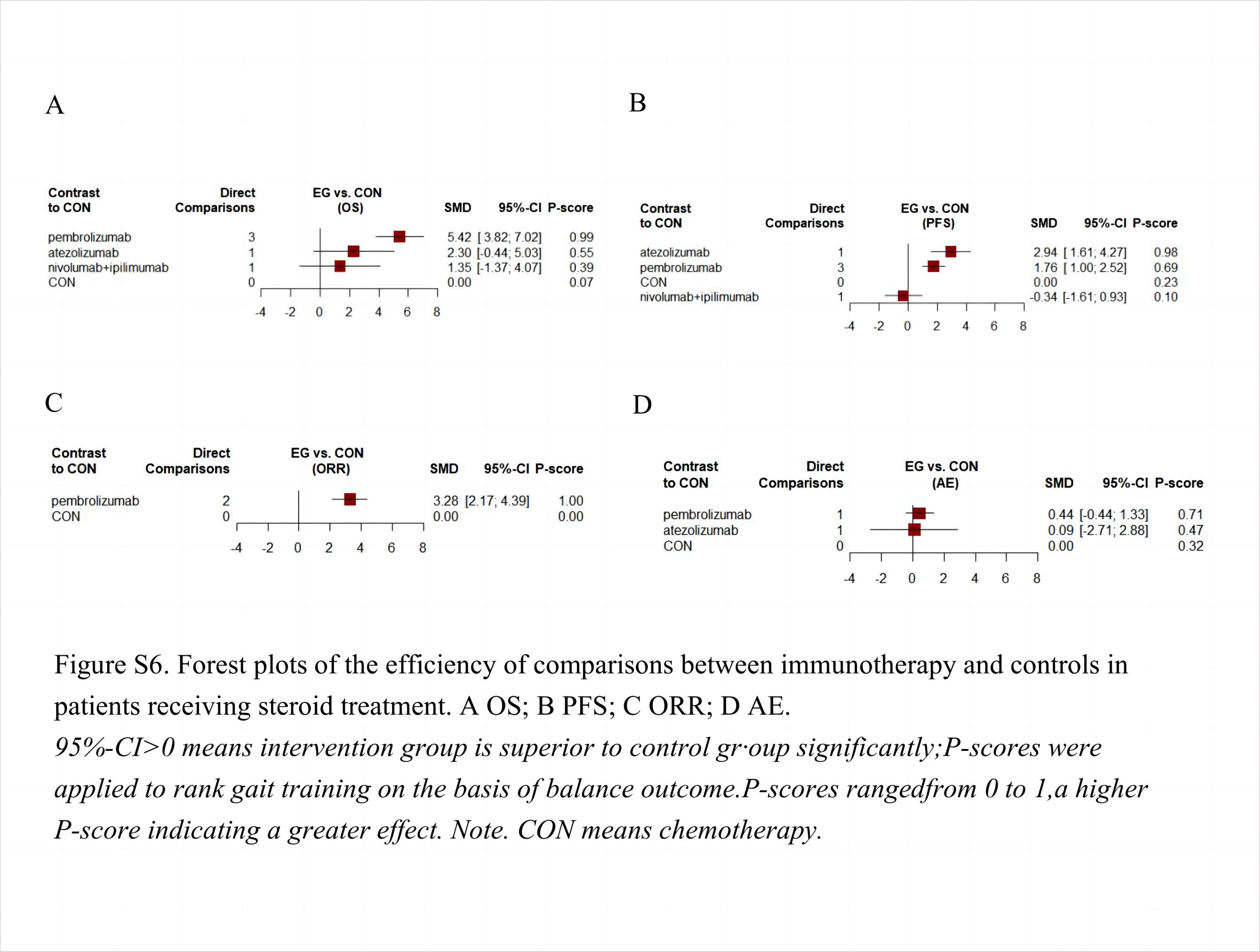

Supplement: Supplementary file 6 — Figure S6 Forest plots of the efficiency of comparisons between immunotherapy and controls in patients receiving steroid treatment. (A) OS; (B) PFS; (C) ORR; (D) AE. 95% CI > 0 means the intervention group is superior to the control group significantly. p‐scores were applied to rank gait training on the basis of balance outcome. p‐comes ranged from 0 to 1, a higher p‐score indicating a greater effect. CON, chemotherapy. [file CRJ-18-e13823-s001.jpg]
