# Supplementary material for: Comparative Efficacy and Safety of Immunotherapy on Non–Small Cell Lung Cancer Patients With Brain Metastases: A Systematic Review and Network Meta‐Analysis
Source: Clin Respir J. 2024 Aug 20;18(8):e13823. doi: 10.1111/crj.13823 (PMC11333852; doi:10.1111/crj.13823)
Supplement: Supplementary file 7 — Table S1 Search strategy for Embase. Table S2. Baseline characteristics of included studies. Table S3. Risk of bias graph of the included studies in this NMA. Table S4. The Newcastle‐Ottawa Scale (NOS) quality assessment of the included studies in this NMA (details). Table S5A. CINeMA confidence rating for OS. Table S5B. CINeMA confidence rating for PFS. Table S5C. CINeMA confidence rating for ORR. Table S5D. CINeMA confidence rating for AE. Table S6A. Metaregression of OS by study type. Table S6B. Metaregression of OS by treatment line. Table S7A. Metaregression of PFS by study type. Table S7B. Metaregression of PFS by treatment line. [file CRJ-18-e13823-s002.docx]

**Table. S1 Search strategy for Embase**

| #1 | 'lung cancer'/exp |
| --- | --- |
| #2 | 'immunotherapy'/exp |
| #3 | atezolizumab:ab,ti OR avelumab:ab,ti OR cemiplimab:ab,ti OR carrelizumab:ab,ti OR durvalumab:ab,ti OR ipilimumab:ab,ti OR nivolumab:ab,ti OR pembrolizumab:ab,ti OR sintilimab:ab,ti OR tislelizumab:ab,ti OR toripalimab:ab,ti OR 'immune checkpoint inhibitor':ab,ti OR 'pd 1':ab,ti OR 'pd l1':ab,ti OR ici:ab,ti OR 'programmed cell death 1 receptor':ab,ti OR 'programmed cell death 1 ligand 1':ab,ti |
| #4 | lung carcinoma':ab,ti OR 'lung neoplasm':ab,ti OR 'pulmonary carcinoma':ab,ti OR 'carcinoma of the lung':ab,ti OR 'pulmonary tumor':ab,ti OR 'pulmonary neoplasm':ab,ti OR 'pulmonary carcinosis':ab,ti OR 'lung carcinogenesis':ab,ti OR 'lung adenocarcinoma':ab,ti OR nsclc:ab,ti OR sclc:ab,ti |
| #5 | #1 OR #4 |
| #6 | #2 OR #3 |
| #7 | (((((((((brain AND metastasis OR brain) AND metastases OR brain) AND metastatic OR cerebral) AND metastases OR cerebral) AND metastatic OR cerebral) AND metastasis OR cranial) AND metastasis OR cns) AND metastasis OR cns) AND metastases OR cns) AND metastatic |
| #8 | #5 AND #6 AND #7 |

**Table. S2 Baseline Characteristics of included studies**

| Author/Year | Type of study | Country | Gender(male) | Age (mean year) | CNS symptoms | Steroid treatment | Treatment line | Intervention  Type | Outcome |
| --- | --- | --- | --- | --- | --- | --- | --- | --- | --- |
| Gadgeel 2020^[29]^ | RCT | USA | IG:45  CG:19 | IG:65 CG:63.5 | no | no | no previous systemic therapies | pembrolizumab | OS，PFS |
| Powell 2021^[30]^ | Retrospective study | Spain | IG:70  CG:36 | IG:63 CG:63.5 | no | no | no previous systemic therapies | pembrolizumab | OS，PFS, ORR, AE |
| Liao 2021^[31]^ | Retrospective study | China | IG:20  CG:21 | 58.4 | not report | not report | undergoing at least first-line treatment | nivolumab | OS，PFS, AE |
| Gadgeel 2019^[32]^ | RCT | USA | IG:34  CG:33 | IG:59 CG:62.5 | no | no | undergoing at least first-line treatment | atezolizumab | OS，PFS, AE |
| Metro 2021^[33]^ | Retrospective study | Italy | IG:4  CG:3 | IG:74 CG:63 | no | not report | no previous systemic therapies | pembrolizumab | OS，PFS |
| Garassino 2019^[34]^ | RCT | USA | IG:73 CG:50 | IG:63.2 CG:62.8 | no | no | no previous systemic therapies | pembrolizumab | OS，PFS, ORR |
| Reck 2021^[35]^ | RCT | Germany | IG:49 CG:43 | IG:65 CG:65 | not report | not report | no previous systemic therapies | nivolumab+ipilimumab | OS |
| Afzal 2018^[36]^ | Retrospective study | USA | IG:13  CG:11 | IG:65.8  CG:63.1 | no | no | no previous systemic therapies | pembrolizumab | ORR |
| Borghaei 2020^[37]^ | RCT | USA | NA | NA | no | no | no previous systemic therapies | nivolumab+ipilimumab | OS，PFS |
| Carbone 2021^[38]^ | RCT | USA | IG:36 CG:35 | IG:65 CG:65 | no | yes | no previous systemic therapies | nivolumab+ipilimumab | OS，PFS, ORR |
| Mansfiel 2019^[39]^ | Retrospective study | USA | IG:73 CG:50 | IG:63.2 CG:62.8 | no | not report | no previous systemic therapies | pembrolizumab | ORR |

*Note. IG: intervention group; CG: control group; NA: not available; CON:chemotherapy.*

**Table. S3 Risk of bias graph of the included included studies in this NMA**

| Study | Random sequence generation | Allocation concealment | Blinding of participants and personnel | Blinding of outcome assessment | Incomplete outcome data | Selective reporting | Other bias | Overall |
| --- | --- | --- | --- | --- | --- | --- | --- | --- |
| Gadgeel 2020 | Low | Low | Low | Low | Low | Low | Low | Low |
| Gadgeel 2019 | Low | High | High | Low | Low | Low | Low | High |
| Garassino 2019 | Low | Unclear | Unclear | Unclear | Low | Low | Low | Moderate |
| Reck 2021 | Low | Low | High | Unclear | Unclear | Unclear | Low | Moderate |
| Borghaei 2020 | Low | Low | High | Unclear | Unclear | Low | Low | Moderate |
| Carbone 2021 | Low | Low | High | Low | Low | Low | Low | Moderate |

**Table. S4 The Newcastle-Ottawa Scale (NOS) quality assessment of the included studies in this NMA (details)**

| Case-Control Star Template | | | | | | | | |
| --- | --- | --- | --- | --- | --- | --- | --- | --- |
| study | Selection of case and controls | | | | Comparability of cases and controls | Exposure | | |
|  | Is the case definition adequate | Representativeness of the cases | Selection of Controls | Definition of Controls | Comparability of cases and controls on the basis of the design or analysis | Ascertainment of exposure | Same method of ascertainment for cases and controls | Non- Response rate |
| Powell 2021 | ＊ |  | ＊ | ＊ | ＊ | ＊ | ＊ | ＊ |
| Liao 2021 | ＊ | ＊ | ＊ | ＊ | ＊ | ＊ | ＊ | ＊ |
| Metro 2021 | ＊ |  | ＊ | ＊ | ＊ | ＊ | ＊ | ＊ |
| Afzal 2018 | ＊ | ＊ | ＊ | ＊ | ＊ | ＊ | ＊ | ＊ |
| Mansfiel 2019 | ＊ |  | ＊ | ＊ |  | ＊ | ＊ | ＊ |

**Table. S5A CINeMA confidence rating for OS**

| Comparison | Number of studies | Within-study bias | Reporting bias | Indirectness | Imprecision | Heterogeneity | Incoherence | Confidence rating |
| --- | --- | --- | --- | --- | --- | --- | --- | --- |
| atezolizumab:CON | 1 | Major concerns | Low risk | No concerns | Major concerns | No concerns | Major concerns | Very low |
| CON:nivolumab | 1 | No concerns | Low risk | No concerns | No concerns | No concerns | Major concerns | Low |
| CON:nivolumab+ipilimumab | 3 | Some concerns | Low risk | No concerns | No concerns | Major concerns | Major concerns | Very low |
| CON:pembrolizumab | 4 | No concerns | Low risk | No concerns | No concerns | Major concerns | Major concerns | Very low |
| atezolizumab:nivolumab | 0 | Some concerns | Low risk | No concerns | No concerns | Major concerns | Major concerns | Very low |
| atezolizumab:nivolumab+ipilimumab | 0 | Some concerns | Low risk | No concerns | Major concerns | No concerns | Major concerns | Very low |
| atezolizumab:pembrolizumab | 0 | Some concerns | Low risk | No concerns | Major concerns | No concerns | Major concerns | Very low |
| nivolumab:nivolumab+ipilimumab | 0 | No concerns | Low risk | No concerns | No concerns | Major concerns | Major concerns | Very low |
| nivolumab:pembrolizumab | 0 | No concerns | Low risk | No concerns | No concerns | Major concerns | Major concerns | Very low |
| nivolumab+ipilimumab:pembrolizumab | 0 | Some concerns | Low risk | No concerns | Major concerns | No concerns | Major concerns | Very low |

*Note: CON means chemotherapy*

**Table. S5B CINeMA confidence rating for PFS**

| Comparison | Number of studies | Within-study bias | Reporting bias | Indirectness | Imprecision | Heterogeneity | Incoherence | Confidence rating |
| --- | --- | --- | --- | --- | --- | --- | --- | --- |
| atezolizumab:CON | 1 | Major concerns | Low risk | No concerns | Major concerns | No concerns | Major concerns | Very low |
| CON:nivolumab | 1 | No concerns | Low risk | No concerns | Major concerns | No concerns | Major concerns | Very low |
| CON:nivolumab+ipilimumab | 2 | Some concerns | Low risk | No concerns | Major concerns | No concerns | Major concerns | Very low |
| CON:pembrolizumab | 4 | No concerns | Low risk | No concerns | Major concerns | No concerns | Major concerns | Very low |
| atezolizumab:nivolumab | 0 | Some concerns | Low risk | No concerns | No concerns | Major concerns | Major concerns | Very low |
| atezolizumab:nivolumab+ipilimumab | 0 | Major concerns | Low risk | No concerns | Major concerns | No concerns | Major concerns | Very low |
| atezolizumab:pembrolizumab | 0 | Some concerns | Low risk | No concerns | Major concerns | No concerns | Major concerns | Very low |
| nivolumab:nivolumab+ipilimumab | 0 | Some concerns | Low risk | No concerns | No concerns | Major concerns | Major concerns | Very low |
| nivolumab:pembrolizumab | 0 | No concerns | Low risk | No concerns | No concerns | Major concerns | Major concerns | Very low |
| nivolumab+ipilimumab:pembrolizumab | 0 | Some concerns | Low risk | No concerns | Major concerns | No concerns | Major concerns | Very low |

*Note: CON means chemotherapy*

**Table. S5C CINeMA confidence rating for ORR**

| Comparison | Number of studies | Within-study bias | Reporting bias | Indirectness | Imprecision | Heterogeneity | Incoherence | Confidence rating |
| --- | --- | --- | --- | --- | --- | --- | --- | --- |
| CON:nivolumab+ipilimumab | 1 | Some concerns | Low risk | No concerns | Major concerns | No concerns | Major concerns | Very low |
| CON:pembrolizumab | 3 | Some concerns | Low risk | No concerns | No concerns | Major concerns | Major concerns | Very low |
| nivolumab+ipilimumab:pembrolizumab | 0 | Some concerns | Low risk | No concerns | No concerns | Major concerns | Major concerns | Very low |

*Note: CON means chemotherapy*

**Table. S5D CINeMA confidence rating for AE**

| Comparison | Number of studies | Within-study bias | Reporting bias | Indirectness | Imprecision | Heterogeneity | Incoherence | Confidence rating |
| --- | --- | --- | --- | --- | --- | --- | --- | --- |
| atezolizumab:CON | 1 | Major concerns | Low risk | No concerns | Major concerns | No concerns | Major concerns | Very low |
| CON:nivolumab | 1 | No concerns | Low risk | No concerns | Major concerns | No concerns | Major concerns | Very low |
| CON:pembrolizumab | 1 | No concerns | Low risk | No concerns | Major concerns | No concerns | Major concerns | Very low |
| atezolizumab:nivolumab | 0 | Some concerns | Low risk | No concerns | Major concerns | Major concerns | Major concerns | Very low |
| atezolizumab:pembrolizumab | 0 | Some concerns | Low risk | No concerns | Major concerns | No concerns | Major concerns | Very low |
| nivolumab:pembrolizumab | 0 | No concerns | Low risk | No concerns | No concerns | Major concerns | Major concerns | Very low |

*Note: CON means chemotherapy*

**Table S6A Meta-regression of OS by study type**

| **Results on the Mean Difference scale** | | | | | |
| --- | --- | --- | --- | --- | --- |
| Iterations = 5010:15000 | | | | | |
| Thinning interval = 10 | | | | | |
| Number of chains = 4 | | | | | |
| Sample size per chain = 1000 | | | | | |
| **Quantiles for each variable:** | | | | | |
|  | 2.5% | 25% | 50% | 75% | 97.5% |
| d.CON.atezolizumab | -5.139 | -0.4303 | 1.36 | 3.039 | 7.468 |
| d.CON.nivolumab | -2.909 | 1.8951 | 3.854 | 5.807 | 11.064 |
| d.CON.nivolumab+ipilimumab | -1.12 | 1.7323 | 2.828 | 3.914 | 6.801 |
| d.CON.pembrolizumab | 1.548 | 3.9339 | 4.847 | 5.719 | 7.937 |
| sd.d | 1.281 | 1.9389 | 2.517 | 3.4 | 5.83 |
| β | -8.409 | -4.2759 | -2.888 | -1.39 | 2.046 |
| **Model fit (residual deviance):** | | | | | |
| Dbar | | pD | | DIC | |
| 9.144289 | | -167.355546 | | -158.211257 | |
| **Regression settings:** | | | | | |
| Estimates at the centering value: study type = 1.333333 | | | | | |

*Note: CON means chemotherapy*

**Table S6B Meta-regression of OS by treatment line**

| **Results on the Mean Difference scale** | | | | | |
| --- | --- | --- | --- | --- | --- |
| Iterations = 5010:15000 | | | | | |
| Thinning interval = 10 | | | | | |
| Number of chains = 4 | | | | | |
| Sample size per chain = 1000 | | | | | |
| **Quantiles for each variable:** | | | | | |
|  | 2.5% | 25% | 50% | 75% | 97.5% |
| d.CON.atezolizumab | -31.50885 | -13.1187 | -3.942 | 2.986 | 12.806 |
| d.CON.nivolumab | -31.80109 | -13.7389 | -4.284 | 2.498 | 13.015 |
| d.CON.nivolumab+ipilimumab | -0.07364 | 3.3991 | 5.598 | 8.38 | 14.062 |
| d.CON.pembrolizumab | 0.58332 | 4.1098 | 6.258 | 8.904 | 14.375 |
| sd.d | 1.51402 | 2.1846 | 2.774 | 3.625 | 5.987 |
| β | -11.2049 | -0.4061 | 6.89 | 17.48 | 37.663 |
| **Model fit (residual deviance):** | | | | | |
| Dbar | | pD | | DIC | |
| 9.016044 | | -2200.974357 | | -2191.958313 | |
| **Regression settings:** | | | | | |
| Estimates at the centering value: study type =1.222222 | | | | | |

*Note: CON means chemotherapy*

**Table S7A Meta-regression of PFS by study type**

| **Results on the Mean Difference scale** | | | | | |
| --- | --- | --- | --- | --- | --- |
| Iterations = 5010:15000 | | | | | |
| Thinning interval = 10 | | | | | |
| Number of chains = 4 | | | | | |
| Sample size per chain = 1000 | | | | | |
| **Quantiles for each variable:** | | | | | |
|  | 2.5% | 25% | 50% | 75% | 97.5% |
| d.CON.atezolizumab | -4.137 | 0.5245 | 2.562 | 4.5453 | 9.804 |
| d.CON.nivolumab | -9.816 | -4.949 | -2.9 | -0.8015 | 4.436 |
| d.CON.nivolumab+ipilimumab | -2.511 | 0.8346 | 2.176 | 3.6349 | 7.309 |
| d.CON.pembrolizumab | -1.602 | 0.7701 | 1.727 | 2.6481 | 5.222 |
| sd.d | 1.424 | 2.1895 | 2.841 | 3.7354 | 5.292 |
| β | -6.472 | -2.4702 | -1.006 | 0.3752 | 3.498 |
| **Model fit (residual deviance):** | | | | | |
| Dbar | | pD | | DIC | |
| 7.942698 | | -18.468157 | | -10.525459 | |
| **Regression settings:** | | | | | |
| Estimates at the centering value: study type = 1.375 | | | | | |

*Note: CON means chemotherapy*

**Table S7B Meta-regression of PFS by treatment line**

| **Results on the Mean Difference scale** | | | | | |
| --- | --- | --- | --- | --- | --- |
| Iterations = 5010:15000 | | | | | |
| Thinning interval = 10 | | | | | |
| Number of chains = 4 | | | | | |
| Sample size per chain = 1000 | | | | | |
| **Quantiles for each variable:** | | | | | |
|  | 2.5% | 25% | 50% | 75% | 97.5% |
| d.CON.atezolizumab | -8.275 | -0.1886 | 3.7207 | 7.749 | 19.487 |
| d.CON.nivolumab | -15.035 | -6.7127 | -2.7089 | 1.559 | 13.439 |
| d.CON.nivolumab+ipilimumab | -4.133 | 0.3548 | 2.3241 | 4.012 | 7.963 |
| d.CON.pembrolizumab | -4.389 | -0.3557 | 1.2102 | 2.716 | 6.058 |
| sd.d | 1.398 | 2.0798 | 2.6643 | 3.524 | 5.184 |
| β | -19.486 | -5.2929 | -0.9096 | 2.808 | 11.845 |
| **Model fit (residual deviance):** | | | | | |
| Dbar | | pD | | DIC | |
| 7.979701 | | -42.919514 | | -34.939813 | |
| **Regression settings:** | | | | | |
| Estimates at the centering value: study type = 1.25 | | | | | |

*Note: CON means chemotherapy*
